# Food allergy and anaphylaxis – 2055: Slow specific oral tolerance induction in children with hen’s egg allergy. 3-days on / 4 days off schedule

**DOI:** 10.1186/1939-4551-6-S1-P138

**Published:** 2013-04-23

**Authors:** Kentaro Mikami, Norifumi Ogawa, Norifumi Ogawa, Sho Mimura, Akihiro Oshiba, Takeshi Noma

**Affiliations:** 1 Department of Pediatrics, Chiba Aiyukai Memorial Hospital, Nagareyama, Japan; 2 Department of Pediatrics, Kitasato University School of Medicine, Sagamihara, Japan; 3 Department of Pediatrics, Kawaguchi Municipal Medical Center, Kawaguchi, Japan; 4 Department of Pediatrics, Tokyo Kousei-Nenkin Hospital, Tokyo, Japan

## Background

Specific oral tolerance induction (SOTI) therapy has been used for treat children with food allergy. Patients usually intake food allergens every day in slow-SOTI protocol, but other protocols have not yet been evaluated.

## Objective

The aim of this study is to evaluate the efficacy of SOTI with 3-days on/4-days off schedule per one week for children with hen’s egg allergy.

## Methods

Seven children aged 9 months to 6.6 years (median 1.7 years) with hen’s egg allergy were performed open oral food challenge tests with boiled hen’s egg to define the threshold dose. Subjects underwent SOTI in which they intake boiled hen’s egg at home 3 days every week. The dose was increased every 1 to 2 weeks from approximately one fourth of the threshold dose to 60g. Clinical response and immunologic changes before and after SOTI were evaluated.

## Results

Six of 7 subjects (85.7%) could intake higher doses of boiled egg more than the threshold doses. It took 30 to 121 days (median 51 days). Serum total immunoglobulin E (IgE) changed from 297 IU/ml (mean of six patients) to 294 IU/ml, egg white-specific immunoglobulin E (sIgE) increased from 10.1 UA/ml to 16.1 UA/ml, ovomucoid sIgE increased from 4.2 UA/ml to 6.7 UA/ml, peripheral eosinophils counts decreased from 550/μl to 472μ/l , and wheal size in skin prick test decreased from 4.4mm (mean diamiter) to 2.7mm after SOTI.

## Conclusions

Three-days on/4-days off schedule in slow SOTI is comparable to everyday schedule for patients with hen’s egg allergy.

